# 4-Anilino-3-nitro-*N*-phenyl­benzamide

**DOI:** 10.1107/S1600536810043849

**Published:** 2010-11-24

**Authors:** Guihua Chen, Jian Yan

**Affiliations:** a School of Pharmaceutical and Chemical Engineering, Taizhou University, Linhai 317000, People’s Republic of China; b Ningbo International Investment Consulting Co. Ltd, Taizhou University, Ningbo 315010, People’s Republic of China

## Abstract

In the title compound, C_19_H_15_N_3_O_3_, the anilino and benzamide rings make dihedral angles of 10.66 (16) and 50.39 (16)°, respectively, with the nitro-substituted benzene ring. The nitro group is slightly twisted by 11.49 (17)° with respect to the attached benzene ring. There is an intra­molecular N—H⋯O hydrogen bond forming an *S*(6) ring. In the crystal, weak inter­molecular N—H⋯O and C—H⋯O hydrogen bonds link the mol­ecules into a chain parallel to the *b* axis. Futhermore, weak slipped π–π inter­actions [centroid–centroid distance = 3.819 (2) Å, inter­planar distance = 3.567 Å and offset angle [how is the offset angle defined?] = 21°] between the anilino ring and its symmetry-related counterpart may help to stabilize the packing.

## Related literature

For the synthesis of the title compound, see: Schelz & Inst (1978 ▶). For related structures, see: McWilliam *et al.* (2001 ▶); Li, Liu *et al.* (2009 ▶); Li, Wu *et al.* (2009 ▶). For discussion of hydrogen bonding, see: Etter *et al.* (1990 ▶); Bernstein *et al.* (1995 ▶).
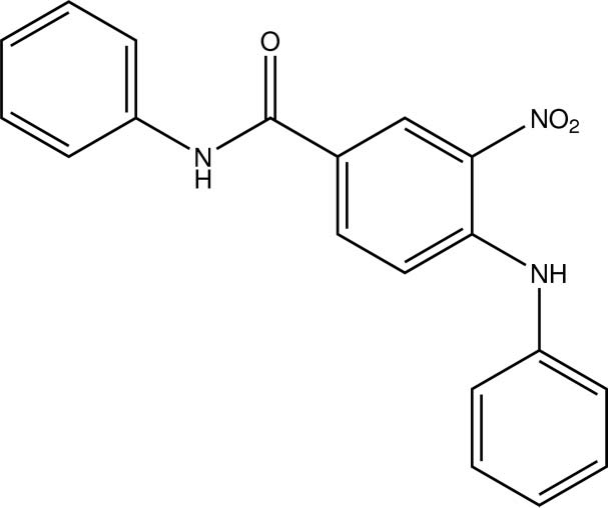


## Experimental

### 

#### Crystal data

C_19_H_15_N_3_O_3_
*M* *_r_* = 333.34
Triclinic, 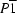

*a* = 7.7930 (16) Å
*b* = 8.1580 (16) Å
*c* = 12.788 (3) Å
α = 84.73 (3)°
β = 83.82 (3)°
γ = 73.58 (3)°
*V* = 773.7 (3) Å^3^
*Z* = 2
Mo *K*α radiation
μ = 0.10 mm^−1^
*T* = 293 K
0.30 × 0.20 × 0.10 mm

#### Data collection

Enraf–Nonius CAD-4 diffractometer
Absorption correction: ψ scan (North *et al.*, 1968 ▶) *T*_min_ = 0.971, *T*_max_ = 0.990
3037 measured reflections
2809 independent reflections
1913 reflections with *I* > 2σ(*I*)
*R*_int_ = 0.069
3 standard reflections every 200 reflections  intensity decay: 1%

#### Refinement

*R*[*F* ^2^ > 2σ(*F* ^2^)] = 0.058
*wR*(*F* ^2^) = 0.173
*S* = 1.07
2809 reflections
226 parameters
H-atom parameters constrained
Δρ_max_ = 0.20 e Å^−3^
Δρ_min_ = −0.27 e Å^−3^

### 

Data collection: *CAD-4 Software* (Enraf–Nonius, 1989 ▶); cell refinement: *CAD-4 Software*; data reduction: *XCAD4* (Harms & Wocadlo, 1995 ▶); program(s) used to solve structure: *SHELXS97* (Sheldrick, 2008 ▶); program(s) used to refine structure: *SHELXL97* (Sheldrick, 2008 ▶); molecular graphics: *ORTEPIII* (Burnett & Johnson, 1996 ▶), *ORTEP-3 for Windows* (Farrugia, 1997 ▶) and *PLATON* (Spek, 2009 ▶); software used to prepare material for publication: *SHELXL97*.

## Supplementary Material

Crystal structure: contains datablocks global, I. DOI: 10.1107/S1600536810043849/dn2614sup1.cif

Structure factors: contains datablocks I. DOI: 10.1107/S1600536810043849/dn2614Isup2.hkl

Additional supplementary materials:  crystallographic information; 3D view; checkCIF report

## Figures and Tables

**Table 1:** Hydrogen-bond geometry (Å, °)

*D*—H⋯*A*	*D*—H	H⋯*A*	*D*⋯*A*	*D*—H⋯*A*
N1—H1⋯O3^i^	0.86	2.39	3.198 (3)	156
N3—H3⋯O2	0.86	2.04	2.649 (3)	127
C5—H5*A*⋯O3^i^	0.93	2.47	3.305 (4)	150
C9—H9*A*⋯O3^i^	0.93	2.51	3.416 (4)	165
C1—H1*B*⋯O1	0.93	2.26	2.851 (4)	121

Symmetry code: (i) 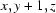.

**Fig. 1.**
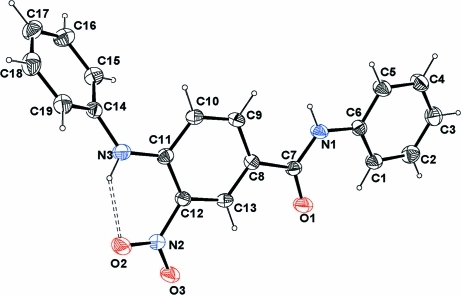
The molecular structure of (I), showing the atom-labeling scheme. Displacement ellipsoids are drawn at the 30% probability level. H atoms are represented as small spheres of arbitrary radii. Hydrogen bond is shown as dashed line.

**Fig. 2.**
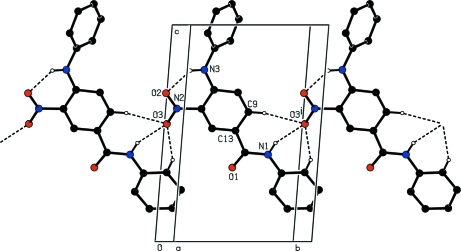
A partial packing view of (I) showing the infinite chain formed by N-H···O and C-H···O hydrogen bonds. H atoms not involved in hydrogen bondings have been omitted for clarity. [Symmetry code: (i) x, 1+y, z]

## supplementary crystallographic information

### Comment

The molecule of the title compound, C_19_H_15_N_3_O_3_, is markedly non-planar, the benzamide (C1 to C6) and the phenyl amino (C14 to C19) rings make dihedral of 10.66 (16)° and 50.39 (16)° respectively, with the nitro substituted phenyl ring (C8 to C13) (Fig. 1). The nitro group is slightly twisted with respect to the phenyl ring by 11.49 (17)°. The bond lengths and bond angles agree with related structures (Li, Liu *et al.*, 2009; Li, Wu *et al.*, 2009; McWilliam *et al.*, 2001).

There is an intramolecular N-H···O hydrogen bond forming an S(6) ring (Etter *et al.*, 1990; Bernstein *et al.*, 1995) whereas weak intermolecular N-H···O and C-H···O hydrogen bonds link the molecules into a chain parallel to the b axis (Table 1, Fig. 2). Futhermore, weak slippest π-π interaction (centroid to centroid = 3.819 (2)Å, interplanar distance = 3.567 and offset angle of 21°) between the C14–C19 phenyl ring and its symmetry related (symmetry code: (i) 1-x, 1-y, 2-z) may help in stabilizing the packing.

### Experimental

4-chloro-3-nitrobenzamide (4.5 g, 0.022 mol)was heat in 10 ml fresh distilled aniline for 18 h at 403 K. After reaction completed (TLC control) was added 50 ml e thanol, at room temperature. The red precipitate was sucked, washed with cold ethanol(2*15 ml), dried over sodium sulfate and gave 5.5 g(74%) (Schelz & Inst,1978). Pure compound (I) was obstained by crystallizing from methanol. Crystals of (I) suitable for X-ray diffraction were obstained by slow evaporation of an methanol solution.

### Refinement

H atoms were positioned geometrically, with C—H = 0.93 Å for aromatic H, and constrained to ride on their parent atoms, with *U*_iso_(H) = 1.2*U*_eq_(C).

### Crystal data

**Table:**

C_19_H_15_N_3_O_3_	*Z* = 2
*M**_r_* = 333.34	*F*(000) = 348
Triclinic, *P*1	*D*_x_ = 1.431 Mg m^−^^3^
Hall symbol: -P 1	Mo *K*α radiation, λ = 0.71073 Å
*a* = 7.7930 (16) Å	Cell parameters from 25 reflections
*b* = 8.1580 (16) Å	θ = 9–13°
*c* = 12.788 (3) Å	µ = 0.10 mm^−^^1^
α = 84.73 (3)°	*T* = 293 K
β = 83.82 (3)°	Block, colourless
γ = 73.58 (3)°	0.30 × 0.20 × 0.10 mm
*V* = 773.7 (3) Å^3^	

### Data collection

**Table:**

Enraf–Nonius CAD-4 diffractometer	1913 reflections with *I* > 2σ(*I*)
Radiation source: fine-focus sealed tube	*R*_int_ = 0.069
graphite	θ_max_ = 25.3°, θ_min_ = 1.6°
ω/2θ scans	*h* = 0→9
Absorption correction: ψ scan (North *et al.*, 1968)	*k* = −9→9
*T*_min_ = 0.971, *T*_max_ = 0.990	*l* = −15→15
3037 measured reflections	3 standard reflections every 200 reflections
2809 independent reflections	intensity decay: 1%

### Refinement

**Table:**

Refinement on *F*^2^	Primary atom site location: structure-invariant direct methods
Least-squares matrix: full	Secondary atom site location: difference Fourier map
*R*[*F*^2^ > 2σ(*F*^2^)] = 0.058	Hydrogen site location: inferred from neighbouring sites
*wR*(*F*^2^) = 0.173	H-atom parameters constrained
*S* = 1.07	*w* = 1/[σ^2^(*F*_o_^2^) + (0.0726*P*)^2^ + 0.3362*P*] where *P* = (*F*_o_^2^ + 2*F*_c_^2^)/3
2809 reflections	(Δ/σ)_max_ < 0.001
226 parameters	Δρ_max_ = 0.20 e Å^−^^3^
0 restraints	Δρ_min_ = −0.27 e Å^−^^3^

### Special details

**Table:**

Geometry. All esds (except the esd in the dihedral angle between two l.s. planes) are estimated using the full covariance matrix. The cell esds are taken into account individually in the estimation of esds in distances, angles and torsion angles; correlations between esds in cell parameters are only used when they are defined by crystal symmetry. An approximate (isotropic) treatment of cell esds is used for estimating esds involving l.s. planes.
Refinement. Refinement of *F*^2^ against ALL reflections. The weighted *R*-factor *wR* and goodness of fit *S* are based on *F*^2^, conventional *R*-factors *R* are based on *F*, with *F* set to zero for negative *F*^2^. The threshold expression of *F*^2^ > σ(*F*^2^) is used only for calculating *R*-factors(gt) *etc*. and is not relevant to the choice of reflections for refinement. *R*-factors based on *F*^2^ are statistically about twice as large as those based on *F*, and *R*- factors based on ALL data will be even larger.

### Fractional atomic coordinates and isotropic or equivalent isotropic displacement parameters (Å^2^)

**Table:**

	*x*	*y*	*z*	*U*_iso_*/*U*_eq_	
O1	0.1624 (3)	0.4949 (3)	0.34191 (17)	0.0597 (7)	
O2	0.4401 (3)	−0.0652 (3)	0.68488 (17)	0.0595 (7)	
O3	0.3047 (3)	−0.0231 (3)	0.54356 (17)	0.0552 (6)	
N1	0.1661 (3)	0.7456 (3)	0.40167 (18)	0.0439 (6)	
H1	0.1892	0.7898	0.4552	0.053*	
N2	0.3633 (3)	0.0321 (3)	0.61399 (18)	0.0408 (6)	
N3	0.4299 (3)	0.2000 (3)	0.79278 (18)	0.0442 (6)	
H3	0.4810	0.0922	0.7892	0.053*	
C1	0.0361 (4)	0.8190 (4)	0.2304 (2)	0.0442 (7)	
H1B	0.0293	0.7078	0.2265	0.053*	
C2	−0.0237 (4)	0.9424 (4)	0.1516 (2)	0.0525 (8)	
H2A	−0.0742	0.9143	0.0955	0.063*	
C3	−0.0102 (5)	1.1066 (4)	0.1543 (3)	0.0562 (9)	
H3B	−0.0473	1.1875	0.0993	0.067*	
C4	0.0583 (5)	1.1499 (4)	0.2389 (3)	0.0535 (8)	
H4A	0.0663	1.2609	0.2420	0.064*	
C5	0.1152 (4)	1.0284 (4)	0.3193 (2)	0.0486 (8)	
H5A	0.1601	1.0588	0.3769	0.058*	
C6	0.1066 (4)	0.8620 (4)	0.3157 (2)	0.0386 (7)	
C7	0.1915 (4)	0.5740 (3)	0.4114 (2)	0.0377 (7)	
C8	0.2585 (4)	0.4838 (3)	0.5121 (2)	0.0372 (7)	
C9	0.2942 (4)	0.5618 (4)	0.5980 (2)	0.0436 (7)	
H9A	0.2794	0.6794	0.5932	0.052*	
C10	0.3501 (4)	0.4676 (4)	0.6883 (2)	0.0431 (7)	
H10A	0.3721	0.5233	0.7435	0.052*	
C11	0.3756 (4)	0.2896 (3)	0.7005 (2)	0.0364 (6)	
C12	0.3430 (4)	0.2137 (3)	0.6127 (2)	0.0361 (6)	
C13	0.2860 (4)	0.3091 (3)	0.5223 (2)	0.0372 (6)	
H13A	0.2654	0.2539	0.4664	0.045*	
C14	0.4108 (4)	0.2654 (3)	0.8927 (2)	0.0371 (7)	
C15	0.2639 (4)	0.3943 (4)	0.9262 (2)	0.0450 (7)	
H15A	0.1758	0.4464	0.8809	0.054*	
C16	0.2481 (4)	0.4458 (4)	1.0272 (2)	0.0537 (8)	
H16A	0.1494	0.5340	1.0490	0.064*	
C17	0.3752 (5)	0.3695 (5)	1.0967 (2)	0.0570 (9)	
H17A	0.3623	0.4045	1.1650	0.068*	
C18	0.5225 (5)	0.2398 (5)	1.0630 (3)	0.0563 (9)	
H18A	0.6098	0.1869	1.1088	0.068*	
C19	0.5402 (4)	0.1891 (4)	0.9619 (2)	0.0454 (7)	
H19A	0.6401	0.1025	0.9397	0.055*	

### Atomic displacement parameters (Å^2^)

**Table:**

	*U*^11^	*U*^22^	*U*^33^	*U*^12^	*U*^13^	*U*^23^
O1	0.0943 (18)	0.0402 (12)	0.0486 (13)	−0.0195 (12)	−0.0183 (12)	−0.0065 (10)
O2	0.0869 (18)	0.0349 (12)	0.0525 (13)	−0.0051 (11)	−0.0210 (12)	0.0004 (10)
O3	0.0802 (17)	0.0388 (12)	0.0546 (14)	−0.0245 (11)	−0.0164 (12)	−0.0064 (10)
N1	0.0598 (16)	0.0353 (13)	0.0422 (14)	−0.0165 (12)	−0.0180 (12)	−0.0038 (11)
N2	0.0497 (15)	0.0350 (13)	0.0375 (13)	−0.0117 (11)	−0.0010 (12)	−0.0049 (11)
N3	0.0540 (16)	0.0363 (13)	0.0406 (14)	−0.0066 (11)	−0.0099 (12)	−0.0061 (11)
C1	0.0492 (18)	0.0436 (17)	0.0429 (17)	−0.0158 (14)	−0.0079 (14)	−0.0048 (13)
C2	0.055 (2)	0.058 (2)	0.0457 (18)	−0.0143 (16)	−0.0132 (15)	−0.0045 (15)
C3	0.064 (2)	0.0492 (19)	0.052 (2)	−0.0109 (16)	−0.0117 (16)	0.0071 (15)
C4	0.065 (2)	0.0389 (17)	0.059 (2)	−0.0170 (16)	−0.0149 (17)	0.0041 (15)
C5	0.059 (2)	0.0436 (17)	0.0498 (18)	−0.0203 (15)	−0.0182 (15)	−0.0015 (14)
C6	0.0376 (15)	0.0391 (16)	0.0409 (16)	−0.0136 (13)	−0.0034 (12)	−0.0023 (12)
C7	0.0404 (16)	0.0384 (16)	0.0366 (15)	−0.0134 (13)	−0.0020 (12)	−0.0084 (12)
C8	0.0412 (16)	0.0349 (15)	0.0379 (15)	−0.0145 (12)	−0.0020 (13)	−0.0029 (12)
C9	0.0564 (19)	0.0315 (15)	0.0464 (17)	−0.0179 (14)	0.0005 (14)	−0.0080 (13)
C10	0.0555 (19)	0.0429 (17)	0.0384 (16)	−0.0231 (14)	−0.0077 (14)	−0.0070 (13)
C11	0.0345 (15)	0.0395 (15)	0.0358 (15)	−0.0110 (12)	0.0002 (12)	−0.0063 (12)
C12	0.0391 (16)	0.0310 (14)	0.0385 (15)	−0.0118 (12)	0.0032 (12)	−0.0057 (12)
C13	0.0437 (16)	0.0351 (15)	0.0360 (15)	−0.0135 (12)	−0.0043 (12)	−0.0094 (12)
C14	0.0410 (16)	0.0370 (15)	0.0372 (15)	−0.0165 (13)	−0.0052 (13)	−0.0013 (12)
C15	0.0409 (17)	0.0485 (18)	0.0460 (18)	−0.0114 (14)	−0.0071 (14)	−0.0048 (14)
C16	0.052 (2)	0.059 (2)	0.0501 (19)	−0.0150 (16)	0.0036 (16)	−0.0168 (16)
C17	0.072 (2)	0.072 (2)	0.0406 (18)	−0.039 (2)	−0.0055 (17)	−0.0085 (16)
C18	0.058 (2)	0.070 (2)	0.0489 (19)	−0.0291 (18)	−0.0169 (16)	0.0079 (17)
C19	0.0445 (17)	0.0489 (18)	0.0453 (17)	−0.0165 (14)	−0.0077 (14)	0.0009 (14)

### Geometric parameters (Å, °)

**Table:**

O1—C7	1.220 (3)	C7—C8	1.492 (4)
O2—N2	1.236 (3)	C8—C13	1.377 (4)
O3—N2	1.225 (3)	C8—C9	1.408 (4)
N1—C7	1.353 (3)	C9—C10	1.368 (4)
N1—C6	1.412 (3)	C9—H9A	0.9300
N1—H1	0.8600	C10—C11	1.405 (4)
N2—C12	1.443 (3)	C10—H10A	0.9300
N3—C11	1.371 (3)	C11—C12	1.410 (4)
N3—C14	1.407 (3)	C12—C13	1.374 (4)
N3—H3	0.8600	C13—H13A	0.9300
C1—C2	1.377 (4)	C14—C15	1.377 (4)
C1—C6	1.387 (4)	C14—C19	1.387 (4)
C1—H1B	0.9300	C15—C16	1.377 (4)
C2—C3	1.377 (4)	C15—H15A	0.9300
C2—H2A	0.9300	C16—C17	1.378 (4)
C3—C4	1.372 (4)	C16—H16A	0.9300
C3—H3B	0.9300	C17—C18	1.383 (5)
C4—C5	1.378 (4)	C17—H17A	0.9300
C4—H4A	0.9300	C18—C19	1.374 (4)
C5—C6	1.383 (4)	C18—H18A	0.9300
C5—H5A	0.9300	C19—H19A	0.9300
			
C7—N1—C6	128.9 (2)	C10—C9—C8	121.1 (3)
C7—N1—H1	115.6	C10—C9—H9A	119.5
C6—N1—H1	115.6	C8—C9—H9A	119.5
O3—N2—O2	121.0 (2)	C9—C10—C11	122.4 (3)
O3—N2—C12	119.0 (2)	C9—C10—H10A	118.8
O2—N2—C12	120.0 (2)	C11—C10—H10A	118.8
C11—N3—C14	127.0 (2)	N3—C11—C10	120.6 (2)
C11—N3—H3	116.5	N3—C11—C12	123.9 (3)
C14—N3—H3	116.5	C10—C11—C12	115.5 (3)
C2—C1—C6	119.4 (3)	C13—C12—C11	121.8 (2)
C2—C1—H1B	120.3	C13—C12—N2	116.5 (2)
C6—C1—H1B	120.3	C11—C12—N2	121.7 (2)
C1—C2—C3	121.3 (3)	C12—C13—C8	122.0 (2)
C1—C2—H2A	119.4	C12—C13—H13A	119.0
C3—C2—H2A	119.4	C8—C13—H13A	119.0
C4—C3—C2	119.4 (3)	C15—C14—C19	119.1 (3)
C4—C3—H3B	120.3	C15—C14—N3	122.6 (3)
C2—C3—H3B	120.3	C19—C14—N3	118.2 (3)
C3—C4—C5	119.8 (3)	C16—C15—C14	119.7 (3)
C3—C4—H4A	120.1	C16—C15—H15A	120.1
C5—C4—H4A	120.1	C14—C15—H15A	120.1
C4—C5—C6	121.1 (3)	C15—C16—C17	121.5 (3)
C4—C5—H5A	119.5	C15—C16—H16A	119.3
C6—C5—H5A	119.5	C17—C16—H16A	119.3
C5—C6—C1	118.9 (3)	C16—C17—C18	118.7 (3)
C5—C6—N1	117.6 (2)	C16—C17—H17A	120.6
C1—C6—N1	123.4 (2)	C18—C17—H17A	120.6
O1—C7—N1	122.4 (3)	C19—C18—C17	120.1 (3)
O1—C7—C8	120.9 (3)	C19—C18—H18A	119.9
N1—C7—C8	116.7 (2)	C17—C18—H18A	119.9
C13—C8—C9	117.2 (3)	C18—C19—C14	120.8 (3)
C13—C8—C7	117.0 (2)	C18—C19—H19A	119.6
C9—C8—C7	125.8 (2)	C14—C19—H19A	119.6
			
C6—C1—C2—C3	1.9 (5)	N3—C11—C12—C13	179.0 (3)
C1—C2—C3—C4	−2.3 (5)	C10—C11—C12—C13	−1.4 (4)
C2—C3—C4—C5	1.0 (5)	N3—C11—C12—N2	0.6 (4)
C3—C4—C5—C6	0.8 (5)	C10—C11—C12—N2	−179.8 (2)
C4—C5—C6—C1	−1.3 (5)	O3—N2—C12—C13	−10.7 (4)
C4—C5—C6—N1	−179.4 (3)	O2—N2—C12—C13	168.6 (3)
C2—C1—C6—C5	−0.1 (4)	O3—N2—C12—C11	167.7 (3)
C2—C1—C6—N1	178.0 (3)	O2—N2—C12—C11	−12.9 (4)
C7—N1—C6—C5	−171.2 (3)	C11—C12—C13—C8	0.4 (4)
C7—N1—C6—C1	10.7 (5)	N2—C12—C13—C8	178.8 (2)
C6—N1—C7—O1	−0.6 (5)	C9—C8—C13—C12	1.0 (4)
C6—N1—C7—C8	179.1 (3)	C7—C8—C13—C12	−178.3 (2)
O1—C7—C8—C13	0.2 (4)	C11—N3—C14—C15	35.4 (4)
N1—C7—C8—C13	−179.5 (3)	C11—N3—C14—C19	−148.7 (3)
O1—C7—C8—C9	−179.0 (3)	C19—C14—C15—C16	0.3 (4)
N1—C7—C8—C9	1.2 (4)	N3—C14—C15—C16	176.2 (3)
C13—C8—C9—C10	−1.3 (4)	C14—C15—C16—C17	−0.9 (5)
C7—C8—C9—C10	177.9 (3)	C15—C16—C17—C18	0.8 (5)
C8—C9—C10—C11	0.2 (5)	C16—C17—C18—C19	0.0 (5)
C14—N3—C11—C10	22.4 (4)	C17—C18—C19—C14	−0.5 (5)
C14—N3—C11—C12	−158.0 (3)	C15—C14—C19—C18	0.4 (4)
C9—C10—C11—N3	−179.2 (3)	N3—C14—C19—C18	−175.6 (3)
C9—C10—C11—C12	1.2 (4)		

### Hydrogen-bond geometry (Å, °)

**Table:**

*D*—H···*A*	*D*—H	H···*A*	*D*···*A*	*D*—H···*A*
N1—H1···O3^i^	0.86	2.39	3.198 (3)	156
N3—H3···O2	0.86	2.04	2.649 (3)	127
C5—H5A···O3^i^	0.93	2.47	3.305 (4)	150
C9—H9A···O3^i^	0.93	2.51	3.416 (4)	165
C1—H1B···O1	0.93	2.26	2.851 (4)	121

Symmetry codes: (i) *x*, *y*+1, *z*.
